# Raptinal: a powerful tool for rapid induction of apoptotic cell death

**DOI:** 10.1038/s41420-024-02120-1

**Published:** 2024-08-21

**Authors:** Amanda J. Smith, Paul J. Hergenrother

**Affiliations:** Department of Chemistry, Carl R. Woese Institute for Genomic Biology, and Cancer Center at Illinois, University of Illinois at Urbana-Champaign, Urbana, IL 61801 USA

**Keywords:** Apoptosis, Chemical tools

## Abstract

Chemical inducers of apoptosis have been utilized for decades as tools to uncover steps of the apoptotic cascade and to treat various diseases, most notably cancer. While there are several useful compounds available, limitations in potency, universality, or speed of cell death of these pro-apoptotic agents have meant that no single compound is suitable for all (or most) purposes. Raptinal is a recently described small molecule that induces intrinsic pathway apoptosis rapidly and reliably, and consequently, has been utilized in cell culture and whole organisms for a wide range of biological studies. Its distinct mechanism of action complements the current arsenal of cytotoxic compounds, making it useful as a probe for the apoptosis pathway and other cellular processes. The rapid induction of cell death by Raptinal and its widespread commercial availability make it the pro-apoptotic agent of choice for many applications.

## Facts

- Raptinal is a rapid-acting and potent inducer of apoptosis that serves as a unique tool in the study cell death pathways.
- In some contexts, Raptinal can also promote pyroptosis through a caspase-3 and GSDME dependent mechanism.
- Raptinal is tolerated in murine tumor models and shows some efficacy at reducing tumor burden.

## Open questions

- What is the direct molecular target of Raptinal?
- Can the potency of Raptinal be leveraged in a selective manner for cancer treatment?
- Could structural modifications of Raptinal tune out its inhibition of Pannexin-1 activity?

## Introduction

**Fig. 1:**
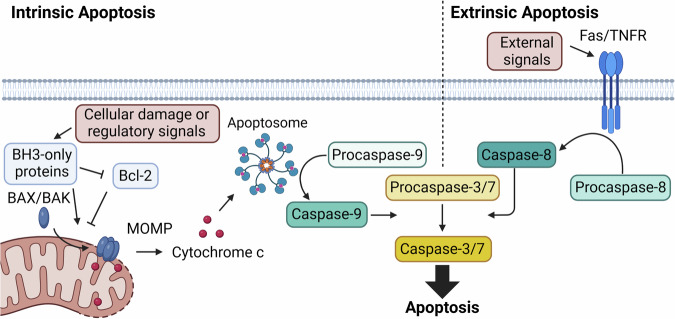
Key events of the intrinsic and extrinsic apoptosis pathways. Created with BioRender.com.

Apoptosis is a mechanism of regulated cell death that is phenotypically characterized by chromatin condensation, breakdown of multiple cellular structures, membrane blebbing, and formation of apoptotic bodies that are subsequently cleared by phagocytosis [1–3]; its initiation is divided into intrinsic and extrinsic pathways. The extrinsic pathway is triggered by external signals binding to death receptors, such as tumor necrosis factor (TNF) binding to the TNF receptor or Fas ligand binding to Fas [3], triggering recruitment of several proteins that lead to formation of active caspase-8 and downstream activation of executioner caspase-3 and -7 from their pro-enzyme forms (Fig. 1) [1, 3]. In contrast, the intrinsic pathway senses perturbations within the cell such as DNA damage or lack of important nutrients or growth factors, but their signals still ultimately lead to activation of executioner caspase-3 and -7. B-cell lymphoma 2 (Bcl-2) family proteins play an important regulatory role in intrinsic apoptosis and include both pro-apoptotic (e.g. BH3-only, BAX, BAK, BOK) and anti-apoptotic (e.g. Bcl-2) members. Bcl-2 and BH3-only respond to various internal stimuli and are anti- and pro-apoptotic regulators (respectively) of BAX and BAK. BAX/BAK/BOK oligomerization and migration to the mitochondrial membrane leads to mitochondrial outer membrane permeabilization (MOMP), cytochrome c release, and subsequent formation of the apoptosome. The apoptosome activates procaspase-9, which is then responsible for activating executioner caspase-3 and -7 from their pro-enzyme forms [1–3]. In both intrinsic and extrinsic apoptosis, activation of procaspase-3 and -7 to caspase-3 and -7 leads to cleavage of scores of proteins, resulting in the observed apoptotic phenotypes [2, 4–6]. Key features of the apoptotic pathway are shown in Fig. 1, and the detailed mechanism of apoptosis has been comprehensively reviewed [3, 7–9].

From the late 1800s to mid-1900s, morphological features of what is now known as apoptosis were observed via microscopy and other imaging techniques [2, 10]. Early studies aimed at elucidating mechanistic details of this pathway relied heavily on biological methods involving gene expression, cloning, and others [10–12]. These initial experiments capitalized on instances of natural cell death and tracked the morphological changes it entails, but as the field advanced, the ability to reproducibly induce apoptosis became essential. As such, the induction of apoptosis by addition or removal of needed nutrients or signaling molecules was common; for example, the removal of interleukin-3 (IL-3) in IL-3 dependent cells drove early discoveries on the roles of Bcl-2 [13] and its effector protein BAX [14] in the signaling cascade, while addition of toxic levels of dATP was used to elucidate the roles of cytochrome c and apoptosome assembly in apoptosis [15–17]. Furthermore, apoptosis triggered by UV irradiation was utilized in the discovery of other important apoptosis regulators, such as smac/DIABLO [18, 19], and larger molecules that activate the extrinsic apoptosis pathway, such as TNFα and anti-Fas antibodies, were leveraged when studying caspase-3-activated substrates [20, 21].

Beyond the previously mentioned approaches, small molecule inducers and inhibitors of apoptosis [22–24] (and other cell death pathways [9, 25–28]) have emerged as vital tools for understanding the mechanistic intricacies and translational potentials of the apoptotic pathway. Methotrexate and staurosporine were early apoptosis-inducing stressors used to show that Bcl-2 could protect cells from apoptosis [29–31], and many other compounds have been instrumental in foundational discoveries about this mode of cell death, with some advancing to the clinic for treating cancer, such as the BH3-mimetic venetoclax [23, 24, 32]. Unsurprisingly, some of the faster-acting compounds like staurosporine and doxorubicin became standard agents for inducing apoptosis [22, 33]. While all of these compounds (Table 1) have been valuable, they are often limited by potency, rate of cell death, or mechanistic targets.

**Table 1:** Common apoptosis inducers used in cell culture.

Compound	Structure	Mechanism of action	Reference
Raptinal		Rapid cytochrome c release from mitochondria	[34]
Methotrexate		Inhibits DHFR and DNA synthesis	[31]
Staurosporine		Kinase inhibitor	[107]
BH3-mimetics (Navitoclax shown)		Inhibits anti-apoptotic Bcl-2 members	[32]
Doxorubicin		Intercalates DNA/inhibits topoisomerase II	[108]
Etoposide		Inhibits topoisomerase II	[108]
Cisplatin		Crosslinks DNA	[111]
PAC-1		Activates procaspase-3 via chelation of labile inhibitory zinc	[118]

Since its discovery in 2015 [34], the small molecule Raptinal has been widely used for rapid and reliable induction of the intrinsic apoptotic pathway, both in cell culture and in whole organisms. Raptinal rapidly induces MOMP, initiating intrinsic apoptosis downstream of BAX/BAK/BOK [35], and has been leveraged in many studies of the apoptotic pathway. Raptinal has also gained utility in the study of pyroptosis due to its integral role in the discovery of caspase-3 mediated and gasdermin-E (GSDME) dependent pyroptosis [36]. This review summarizes current literature on Raptinal’s mechanism of action, its utilization in cell death research, and preliminary insights into its translational potential. Papers utilizing Raptinal are being published at a rapid rate; this review covers publications available through June 2024.

## Raptinal mechanism of action – induction of apoptosis

**Fig. 2:**
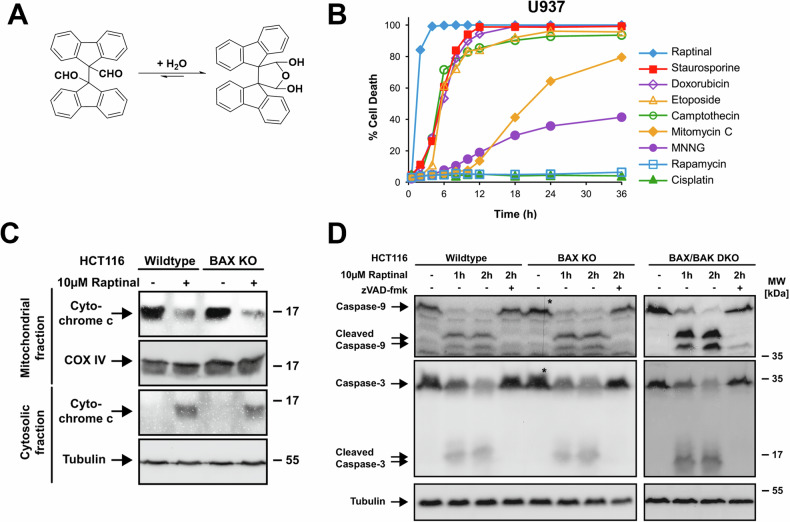
Raptinal rapidly induces apoptosis downstream of BAX/BAK. **A** Structure of Raptinal. **B** Raptinal time to cell death compared to other compounds dosed at 10 μM in U-937 cells. **C** Raptinal at 10 μM induces cytochrome c release independent of BAX/BAK as demonstrated by a 15 min incubation in HCT116 cells. **D** Raptinal at 10 μM induces intrinsic apoptosis independent of BAX/BAK as seen by caspase-9 and caspase-3 activation in wildtype, BAX knockout, and BAX/BAK double knockout HCT116 cells. Panels **A** and **B** were reproduced from Palchaudhuri et al. [34], copyright 2015 CC BY 4.0. Panels **C** and **D** were reproduced from Heimer et al. [35], copyright 2019 CC BY 4.0.

Raptinal, which exists in the dialdehyde form as a solid but is rapidly hydrated in solution (Fig. 2A) [34], has been shown to induce apoptotic cell death across a wide variety of cell types as assessed by a combination of morphological studies and classical markers for apoptosis, such as annexin-V and propidium iodide staining by flow cytometry and caspase-3/7 activation/inhibition assays [34]. To date, there are four major reports with detailed explorations of the mechanism of action (MOA) of Raptinal [34–37]. Raptinal was found to induce apoptosis more rapidly in U-937 cells than a battery of other prominent apoptosis-inducing and anticancer compounds in head-to-head experiments (Fig. 2B) [34], suggesting a different MOA from these other compounds. Initial examination of the MOA revealed that Raptinal treatment of U-937 cells led to rapid release of cytochrome c from the mitochondria and activation of procaspase-9 and -3, and protection from cell death was afforded by the pan-caspase inhibitor QVD-OPh [34] or zVAD-fmk [35].

These results indicate that Raptinal induces intrinsic apoptosis, and in support of this, siRNA knockdown of key genes encoding intrinsic apoptotic proteins reduced sensitivity to Raptinal [34]. In a siRNA screen, knockdown of mRNA encoding apoptosis activating factor 1 (APAF1), CASP3, and CASP9 afforded protection from Raptinal-induced cell death at short timepoints [34, 38]. These unbiased knockdown studies provide strong support for intrinsic apoptotic induction by Raptinal.

Commonly used pro-apoptotic agents affect vital cellular processes, ultimately leading to apoptotic cell death. For example, staurosporine inhibits multiple kinases leading to the induction of apoptosis, and doxorubicin intercalates DNA, inhibiting topoisomerase II and leading to DNA damage which triggers the intrinsic apoptotic pathway. In contrast to these compounds, Raptinal has been shown to act downstream of BAX/BAK/BOK in the apoptotic pathway to elicit cell death [35]. Raptinal treatment of HCT116 cells led to MOMP and cytochrome c release in both wildtype and BAX knockout cells (Fig. 2C) [35]. Accordingly, Raptinal-induced activation of caspases is not altered by BAX knockout or BAX/BAK double knockout as would be expected if Raptinal was triggering apoptosis upstream of BAX/BAK activity (Fig. 2D) [35]. While Raptinal leads to cytochrome c release independent of BAX/BAK in whole cell studies, it does not lead to cytochrome c release from isolated mitochondria; other apoptosis inducers (such as the pro-apoptotic Bcl-2 family member Bid) are able to elicit cytochrome c release in isolated mitochondria [34]. The mechanism of Raptinal-induced MOMP and the exact molecular target of Raptinal is still unknown, as discussed in *Future Directions*.

## Raptinal as a tool compound in the study of apoptosis

Given its ability to quickly induce apoptotic death in a variety of cell types, its distinct MOA compared to other common pro-apoptotic compounds, and its commercial availability from multiple vendors, Raptinal use as a tool compound has been increasing rapidly. Raptinal has been instrumental in advancing the understanding of apoptosis, exploring cancer biology, and providing a reliable means for quantitative induction of cell death [39–44]. In addition to using Raptinal to learn more about apoptosis, it has also been an important tool in the study of other mechanisms of cell death such as pyroptosis [36, 45–48], necrosis [49, 50], and ferroptosis [51]. With Raptinal utilized in over 100 publications, it is impractical to cover everything herein; illustrative examples have been selected where Raptinal has been used in a variety of circumstances.

### Raptinal as a tool in cell death assays

The ability of Raptinal to induce cell death rapidly and quantitatively makes it an ideal control in cell death assays. Raptinal has found general utility as a positive control for cell death in cytotoxicity assays [52–62]. In addition to controls for general cell death, Raptinal has been widely used as a positive control for apoptotic cell death, particularly in quantifying relative caspase activation [63–79]. Given the recent literature on Raptinal-induced pyroptosis (described further below), Raptinal has also been used as a positive control for pyroptotic cell death [79–82], and in multiple other circumstances [83–90].

### Raptinal as a tool for exploring the apoptotic cell death pathway

**Fig. 3:**
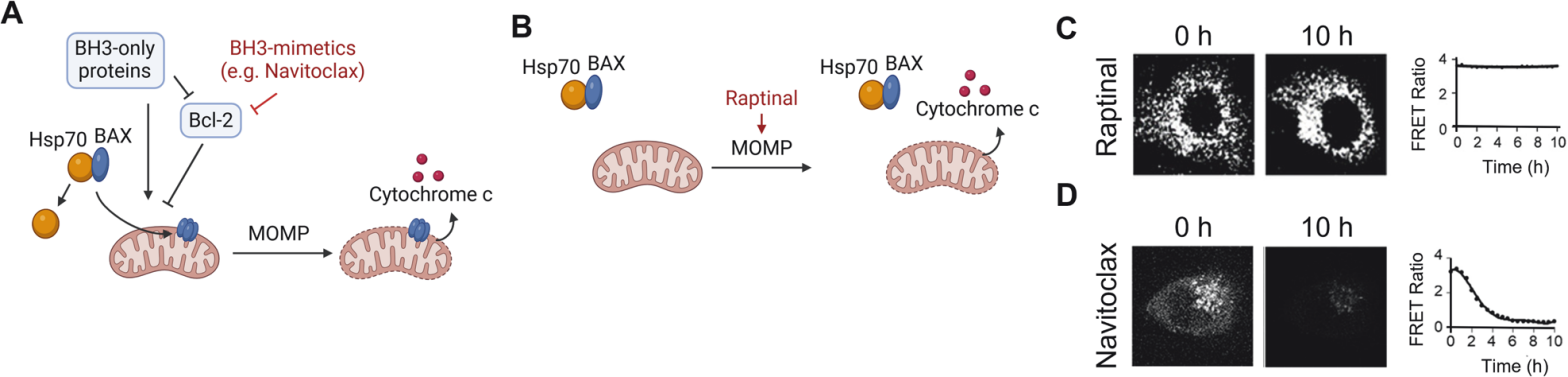
Raptinal as a probe for various roles of BAX/BAK. **A** BH3-mimetics act upstream of BAX dissociation from Hsp70 and migration to the mitochondria, but **B** Raptinal triggers MOMP downstream of BAX. **C** In a FRET-based assay using HeLa cells expressing BAX-F105ANAP and Hsp70-YFP to track BAX association with Hsp70, Raptinal (5 μM) does not cause dissociation of BAX from Hsp70. **D** BH3-mimetic Navitoclax (0.5 μM) results in dissociation of BAX from Hsp70 as seen by loss of FRET signal. Panels **A** and **B** were created with BioRender.com. Panel **C** was reproduced from Park et al. [91] Reprinted with permission from Wiley-VCH Verlag GmbH & Co. KGaA, Weinheim, copyright 2019. Panel **D** was reproduced from Park et al. [92]. Reprinted with permission from American Chemical Society, copyright 2019.

To better understand apoptotic signaling events, it is helpful to have a means to isolate different steps within that pathway. Raptinal’s ability to induce MOMP and cytochrome c release has aided investigations designed to tease apart actions upstream and downstream of these events. For instance, inactive BAX is located in the cytoplasm and associated with Hsp70 and other proteins, but activated BAX dissociates and migrates to the mitochondria (Fig. 3A) [91]. Various stressors, such as ER stress and lysosomal damage, can affect BAX’s dissociation from Hsp70 [91]. As Raptinal acts downstream of BAX, Raptinal treatment demonstrated that disrupting mitochondrial function does not directly contribute to BAX’s dissociation from Hsp70 (Fig. 3B, C) [91]. Other apoptotic inducers, such as the BH3-mimetic navitoclax, are known to activate BAX upstream of MOMP (Fig. 3D) [92] and, therefore, could not be utilized to determine if mitochondrial damage affected BAX’s association with Hsp70.

**Fig. 4:**
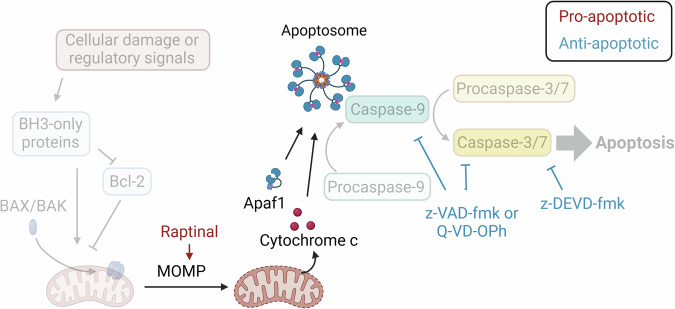
Raptinal leveraged to probe steps of the apoptosis pathway. Pairing Raptinal treatment with a caspase inhibitor (e.g. z-VAD-fmk, Q-VD-OPh, or z-DEVD-fmk) can isolate steps of the apoptotic pathway, specifically cytochrome c release is isolated from upstream BAX/BAK activation and is isolated from downstream caspase activity as seen in studies from Duncan-Lewis, et al. [95] and Renaud, et al. [97] Created with BioRender.com.

The BAX/BAK independence of Raptinal-induced cell death has also been useful in probing how Parkin, an E3 ubiquitin ligase, is involved in determining whether mitochondrial damage leads to the induction of apoptosis or if the damaged mitochondria are simply degraded [93]. Raptinal’s BAX/BAK independence was also important in the investigation of inflammatory responses within the cell that are triggered by MOMP [94]. In another study, Raptinal was employed to elucidate how MOMP alters the feedback between mRNA decay and transcription during apoptosis [95]. By inducing and protecting from apoptosis at various points along the pathway, MOMP was found to be a critical step in causing RNA polymerase II activity to decrease in response to mRNA decay, whereas RNA polymerase II activity would typically increase in response to mRNA decay in healthy cells [95]. Additionally, the structural roles and apoptotic functions of RTN4, CLIMP-63, and DRP1 were probed using BH3 mimetics and Raptinal to help decipher which proteins are critical upstream vs downstream of BAK activation [96]. Raptinal co-dosed with caspase inhibitors allows for ‘isolation’ of this part of the pathway, with cytochrome c release from the mitochondria isolated both from the activation of upstream proteins and from downstream apoptosis (Fig. 4). In one study, Raptinal co-treatment with the pan-caspase inhibitor z-VAD-fmk was used to discover the role of centrosomal protein 131 in mitochondrial homeostasis such that its genetic deletion made the mitochondria more resistant to permeabilization [97]. Treatment with Raptinal and z-VAD-fmk allowed the initial MOMP to be studied without additional mitochondrial permeabilization that occurs due to caspase activation in a positive feedback loop [97]. Thus, with this co-dosing, MOMP and cytochrome c release can be studied independent of the rest of the apoptotic pathway.

### Raptinal as a tool compound in cancer research

**Fig. 5:**
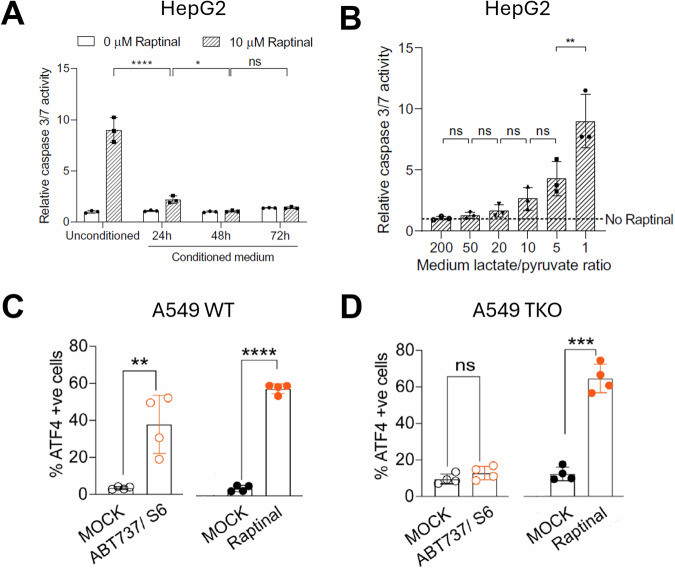
Apoptosis resistance and Raptinal treatment. **A** The Warburg effect alters sensitivity to apoptosis as demonstrated by Raptinal resistance observed in HepG2 cells at 1.5 h treatment when incubated in media previously exposed to Raptinal-treated HepG2 cells (conditioned media) compared to HepG2 cell incubated with fresh media (unconditioned media). **B** In HepG2 cells treated with 10 μM Raptinal for 1.5 h, resistance to Raptinal-induced apoptosis is correlated with lactate to pyruvate ratio in the cell culture media. **C** Increased ATF4 expression correlating to persister cell formation is seen in wild type A549 cells treated with sublethal levels of Raptinal (5 μM) or combination BH3 mimetics ABT737 (1.5 μM) and S6 (3 μM) for 4 h. **D** In BAX/BAK/BOK triple knockout A549 cells, sublethal Raptinal treatment (5 μM) still leads to increased ATF4 expression at 4 h, unlike combination ABT737 (1.5 μM) and S6 (3 μM) treatment at 4 h. Panels **A** and **B** were reproduced from Go et al. [103] copyright 2021 CC BY 4.0. Panels **C** and **D** were reproduced from Kalkavan et al. [104] Reprinted with permission from Elsevier Inc., copyright 2022.

In addition to learning more about apoptosis itself, discoveries utilizing Raptinal have important implications in cancer research. A hallmark of cancer is the evasion of apoptosis [98–100]; thus, a better understanding of apoptosis and cell death can inform treatment. For example, the Warburg effect is an important concept in cancer biology, whereby cancer cells meet their high energy demands by generating ATP quickly through glycolysis and lactic acid fermentation instead of the more efficient route through the citric acid cycle and electron transport chain; this “Warburg effect” can be correlated with resistance to chemotherapy [101, 102]. To corroborate this, it was shown that while HepG2 cells in fresh media are sensitive to Raptinal, cells that are treated in “conditioned media” (media containing supernatant from other growing HepG2 cells) are no longer susceptible to Raptinal (Fig. 5A) [103]. This resistance correlates with the lactate to pyruvate ratio that is altered due to the Warburg effect (Fig. 5B) [103]. The lactate/pyruvate ratio, which is in equilibrium with the NADH/NAD^+^ ratio, affects the activity of c-Jun N-terminal kinases (JNK), a protein that acts downstream of reactive oxygen species (ROS) and promotes expression of apoptotic genes and activation of several BCL-2 proteins [103]. Accordingly, HepG2 cells treated with the JNK inhibitor SP600125 are less susceptible to Raptinal [103]. Thus, developing therapies to correct the lactate/pyruvate ratio in tumors may sensitize resistant cancer cells to pro-apoptotic compounds.

In cancer treatment, it is common that insufficient eradication of the tumor cells by a chemotherapeutic drug leads to regrowth of a more resistant tumor. There are many mechanisms that prevent quantitative cell death induction, such as genetic mutations or upregulation of protective proteins that give a survival advantage to the cells [23]. Recently, it has been shown that when persister cells are generated in the laboratory through sublethal treatment by BH3 mimetics and inoculated into mice, the resulting tumors are more resistant to apoptosis and more likely to metastasize [104]. Persister cells were also able to be generated by sub-lethal treatment of Raptinal, independent of BAX, BAK, and BOK activation and independent of caspase activation. Since Raptinal bypasses BAX, BAK, and BOK in its induction of cell death, they were able to determine that these proteins were not necessary in the development of persistence, but that MOMP and cytochrome c release were the critical steps due to the resulting increase in expression of activating transcription factor 4 (ATF4) which triggers various pro-survival mechanisms (Fig. 5C, D) [104]. Thus, through this study Raptinal helped uncover new potential targets for reversing persister cells in cancer treatments.

## Raptinal’s applications beyond 2D cell culture

While this review has thus far highlighted Raptinal’s utility in 2D cell culture systems, Raptinal has been utilized in other settings as well. Its ability to rapidly induce cell death was utilized in creating scaffolds of extracellular matrices for astrocyte migration assays [105]. Here cells were induced to generate large quantities of extracellular matrix and treated with Raptinal to kill the cells, leaving just the scaffold for further experiments [105]. Raptinal also has proven to be relevant in the study of multicellular systems. Raptinal aided in elucidating the role of epithelial-like cells in the early embryo, where the epithelial cells were able to phagocytize apoptotic cells killed by Raptinal [106]. This study demonstrated Raptinal’s utility in killing cells from both zebrafish and murine embryos [106]. Elsewhere, Raptinal has been shown to induce apoptosis in zebrafish and to be effective in treating murine tumors [34]. The in vivo efficacy of Raptinal will be discussed further in the section on *Raptinal’s translational potential*.

## Raptinal compared to other pro-apoptotic tool compounds

**Fig. 6:**
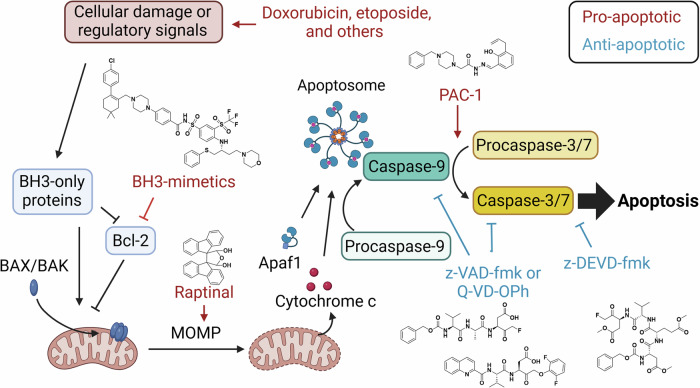
Inducers and inhibitors of apoptosis. Small molecule tool compounds can be used to target different steps of the intrinsic apoptotic pathway. Created with BioRender.com.

Apoptosis is a natural means for the body to clear out cells that are damaged or those that are no longer needed during certain stages of development, and as such, cells possess many sentinels for detecting this damage. Staurosporine is a non-specific kinase inhibitor that has been heavily used for inducing apoptosis in cell culture but has not been suitable for clinical use [107]. For many apoptosis inducers approved for cancer treatment and used in the laboratory, such as doxorubicin, etoposide, and cisplatin, DNA damage is an integral part of triggering apoptosis. By binding DNA and topoisomerase II, etoposide inhibits the ability of topoisomerase II to ligate DNA after detangling its strands [108, 109]. Doxorubicin intercalates with DNA, leading to topoisomerase II inhibition, and doxorubicin can produce further cell damage through generation of ROS [108, 110]. Cisplatin binds DNA, causing damage and interfering with DNA repair, and cisplatin may lead to generation of damaging free radicals [111]. If the cell senses an overwhelming amount of damage, apoptosis will be induced upstream of the Bcl-2 proteins [112]. Guided by knowledge of the apoptosis pathway, BH3-mimetics such as navitoclax and obatoclax have been developed to interfere directly with the anti-apoptotic Bcl-2 members and bypass upstream damage sensors that funnel into the apoptotic cascade [32, 113]. Looking further down the apoptosis pathway, PAC-1 is a clinical drug candidate that activates procaspase-3 to caspase-3 [73, 114–116]. PAC-1 has therefore been useful for probing late stages of the apoptotic pathway [34, 51, 117–121]. As demonstrated in the section *Raptinal as a tool compound in the study of apoptosis*, Raptinal has proven to be a useful chemical tool to induce apoptosis downstream of BAX/BAK but still achieve MOMP. Thus, it is a mechanistically distinct addition to the arsenal of apoptosis inducers (Fig. 6).

Raptinal’s mechanism of action remains distinct from other pro-apoptotic compounds that target the mitochondria. The Hsp70 inhibitor apoptozole (Az) was derivatized to include the mitochondria-targeting triphenylphosphonium (TPP) motif to generate the compound Az-TPP-O3 [122]. Az-TPP-O3 disrupts the binding of the Hsp70 mortalin with p53, and p53 mediated MOMP via BAK. Thus, while AzTPP-O3 is able to act directly within the mitochondria, it is still BAK dependent, unlike Raptinal. Cytotoxicity assays show that 72 h IC_50_ values for Az-TPP-O3 are comparable to 24 h IC_50_ values for Raptinal. Az-TPP-O3 treatment of HeLa, MIA-Paca2, and HL60 cells for 72 h produced IC_50_ values of 0.61, 0.86, and 0.48 μM respectively [122]. In the same cell lines, Raptinal 24 h treatment yields IC_50_ values of 0.6, 1.9, and 2.1 μM, respectively, suggesting approximately equal potency [34]. While Az-TPP-O3 time to cell death was not analyzed, flow cytometry analysis of annexin-V and propidium iodide staining was performed using an 18 h incubation at 2 μM. While this is a longer incubation time than is typically seen in Raptinal assays, it is also a lower compound concentration, and more direct comparisons are needed.

Raptinal stands out among other apoptosis inducers for its accessibility and ability to rapidly induce cell death. Raptinal is commercially available and can be synthesized on a multi-gram scale [34], and its rate of cell death induction makes it a time-efficient control, where cell death can be achieved at a faster rate than even staurosporine and doxorubicin [34]. Thus, Raptinal rounds out the apoptotic arsenal in terms of both mechanism and speed. Other established pro-apoptotic compounds can be leveraged in instances requiring longer time to cell death, such as monitoring the effect of the apoptotic pathway on other cellular processes or for tailoring the cell death control to act in a similar mechanism or on a similar timescale as an experimental compound. Meanwhile, Raptinal is unique in its ability to bypass BAX/BAK/BOK in intrinsic apoptosis, and useful as a control when efficient cell death is required. For further examples:

- Raptinal’s speed of cell death compared to other compounds has also been described in HOS, H1993, SK-MEL-5, Mia PaCa-2, and ES-2 cell lines [34, 51]. In a head-to-head comparison of 10 μM compound treatment of U-937 cells, Raptinal’s time to 50% cell death was only 1.5 h while other fast-acting apoptotic inducers such as staurosporine, etoposide, and doxorubicin required 5 h, 5 h, and 6 h, respectively [34].
- While studying apoptosis induced by *H. pylori*, achieving similar levels of caspase-3 cleavage in gastric epithelial cell lines required 24 h incubation with cisplatin but only 2 h with Raptinal (both at 10 μM); understandably, Raptinal was used throughout the remaining experiments [39].
- Raptinal’s superior speed was also apparent in experiments demonstrating that cell death from *Yersinia* infection is distinct from canonical apoptosis [40]. Here, Raptinal was shown to kill at a similar timescale to the test compound; etoposide at a 15X higher concentration than Raptinal required an additional 16 h incubation to reach the desired endpoint [40].
- Raptinal killed on a similar timescale to experimental studies of nigericin treatment of *casp1* deficient BMDCs; whereas doxorubicin required several more hours [123].
- In a paper studying nuclear expulsion during cell death, it takes staurosporine 25 h to reach the same level of chromatin expansion that Raptinal displays in 5 h, and Raptinal is used throughout many of their experiments [124].
- A study of Hippo kinases shows the importance of MST1 and MST2 in PARP-1 and caspase-3 activation by low concentrations of staurosporine and Raptinal. Western blots compare 3 h Raptinal treatment (3 μM and 10 μM) with 4 h staurosporine (0.5 μM and 2 μM). Variations in timing and dosing limit comparisons; nevertheless, testing both Raptinal and staurosporine as unique inducers of apoptosis validates the study’s findings [125].
- Comparing to another compound that targets the mitochondria, caspase activation and cell death are achieved quicker with Raptinal than the mitochondrial complex I inhibitor rotenone [34, 48].

Assays with Raptinal typically utilize relatively low concentrations, with 10 μM or lower being common. Cytotoxicity of Raptinal has been recorded on short timescales, with 24 h IC_50_ values in the single-digit micromolar range [34, 126]. There is limited head-to-head comparisons of Raptinal with other compounds, but for a general comparison across different publications, 24 h treatment of HeLa cells with Raptinal [34], doxorubicin [127], and cisplatin [127] yield IC_50_ values of 0.6, 1.7, and 77.4 μM, respectively. HepG2 cells treated with Raptinal [126], doxorubicin [128], and 5-flourouricil [126] yield IC_50_ values of 0.62, 11.1, and >100 μM, respectively. The relative potency of drugs is not always realized at 24 h, such as 5-fluororicil (which partially relies on DNA repair mechanisms), so it is hard to conclude how Raptinal compares on longer time scales. However, given its single-micromolar 24 h IC_50_ values, Raptinal is equally if not more potent than many other apoptotic inducers.

## Raptinal mechanism of action – beyond apoptosis

### Inhibition of PANX1 activity

**Fig. 7:**
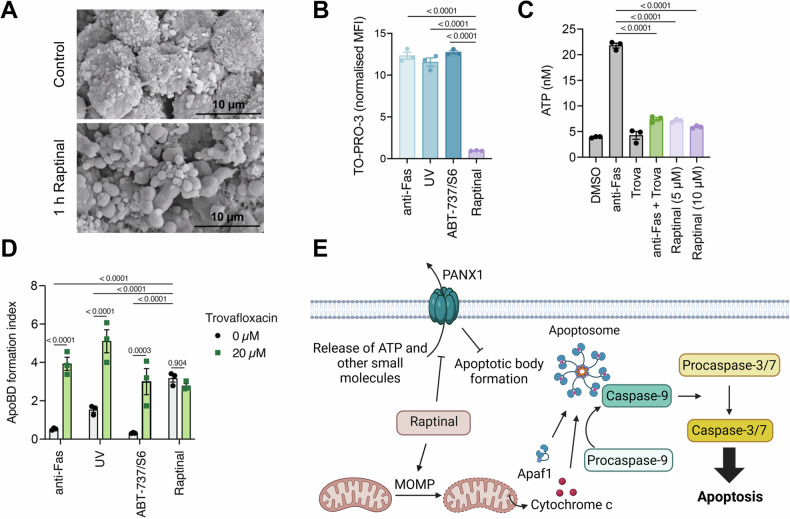
Raptinal inhibits PANX1 activity. **A** Membrane blebbing in U-937 cells with and without 1 h treatment with 10 μM Raptinal. **B** After 4 h of treatment, 10 μM Raptinal causes less uptake of membrane impermeable dye TO-PRO-3 in Jurkat cells relative to treatment with other inducers of apoptosis (anti-Fas (250 ng/mL), UV irradiation (150 mJ cm^−2^), and ABT-737 (5 μM)/S63845 (0.5 μM)). **C** Raptinal treatment of Jurkat cells leads to less ATP release relative to apoptosis inducer anti-FAS (250 ng/mL) and is similar to anti-FAS cotreated with PANX1 inhibitor trovafloxacin (trova) (20 μM). **D** The formation of apoptotic bodies in Jurkat cells after 4 h incubation with 10 μM Raptinal is similar to cotreatment of other apoptosis inducers (anti-Fas (250 ng/mL), UV irradiation (150 mJ cm^−2^), and ABT-737 (5 μM)/S63845 (0.5 μM)) with trovafloxacin. **E** The activity of Raptinal involves both the induction of apoptosis and inhibition of the activity of PANX1. Panel **A** was reproduced from Palchaudhuri et al. [34] copyright 2015 CC BY 4.0. Panels **B** and **C** were reproduced from Santavanond et al. [37] copyright 2024 CC BY 4.0. Panel **E** was created with BioRender.com.

As mentioned previously, phenotypic characteristics of apoptosis include membrane blebbing and the formation of apoptotic bodies. These phenotypes are particularly notable and rapidly appear in U-937 cells treated with Raptinal (Fig. 7A). Considering this, a recent study uncovered a novel role of Raptinal as an inhibitor of the activity of the cell membrane channel Pannexin 1 (PANX1), and Raptinal treatment resembled cotreatment of apoptosis inducers and PANX1 inhibitors in several cell lines [37]. PANX1 is a substrate of caspase-3 and allows the bidirectional flow of small molecules and metabolites, such as ATP, into and out of the cell. Additionally, PANX1 allows the uptake of dyes such as TO-PRO-3, which is leveraged in flow cytometry staining for cell viability. Raptinal treatment prevents the flow of these molecules through PANX1 channels; this result stands in contrast to the effect of other apoptosis inducers including anti-FAS, UV irradiation, and the BH3-mimetic combination ABT-737/S6 (Fig. 7B, C). PANX1 has an inhibitory effect on the formation of apoptotic bodies, and it was found that Raptinal’s inhibition of PANX1 activity promotes apoptotic body formation. While addition of a PANX1 inhibitor (trovafloxacin) increases the apoptotic body formation in cells treated with other apoptosis inducers, the formation of these structures was not augmented in the treatment with Raptinal and PANX1 inhibitor (Fig. 7D). Instead, Raptinal alone resembled the cotreatment of the other apoptotic compounds with the PANX1 inhibitor. In efforts to separate the proapoptotic effects of Raptinal from its inhibition of PANX1 activity, PANX1 was expressed in Hek293T cells with the caspase-3 cleavage site exchanged for a tobacco etch virus (TEV) protease cleavage site. This allowed activation of PANX1 while simultaneously inhibiting caspase activity via Q-VD-OPh, and it demonstrated the effect of Raptinal on PANX1 activity was independent of its ability to induce apoptosis [37]. While this work established the inhibition of PANX1 activity by Raptinal, the exact mechanism of this inhibition has not been elucidated. The mechanism seems to be distinct from other PANX1 inhibitors, such as carbenoxolone and trovafloxacin, which act quickly to inhibit and can be washed out to restore PANX1 activity. Inability to wash out Raptinal and regain PANX1 activity may suggest covalent binding, and delay in PANX1 inhibition by Raptinal may suggest either a metabolite of Raptinal is responsible or that Raptinal acts on an intermediate target that can then inhibit PANX1 [37]. Regardless of the exact mechanism, Raptinal induces apoptosis by inducing MOMP and inhibits the activity of PANX1 (Fig. 7E).

### Caspase-independent cell death induced by Raptinal

**Fig. 8:**
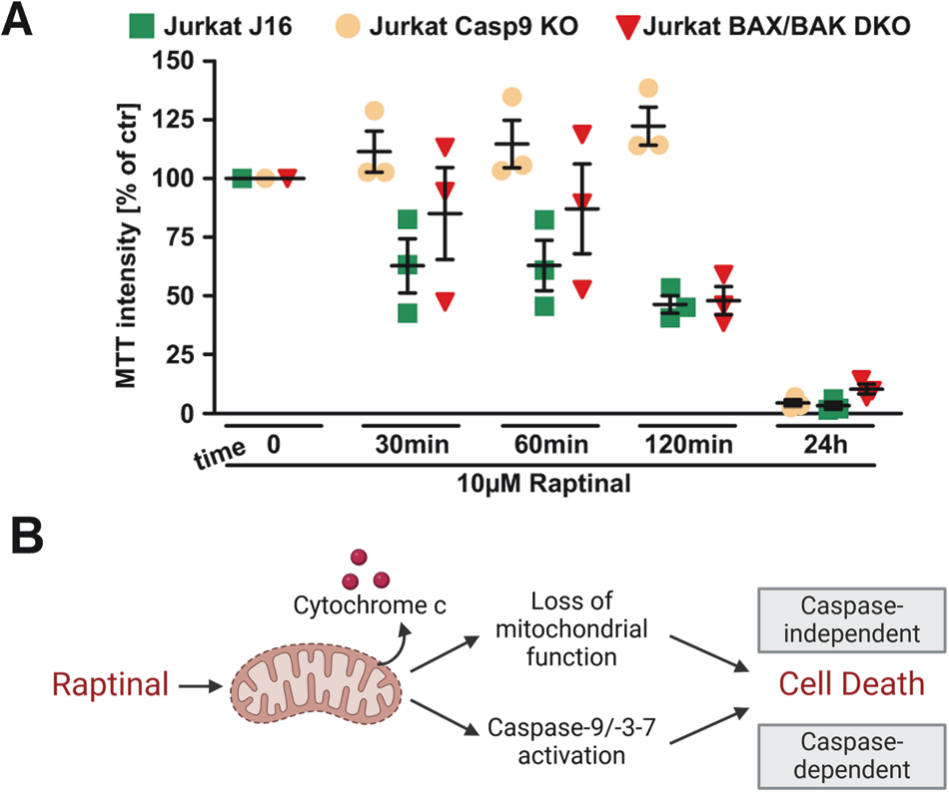
Caspase independent cell death by Raptinal. **A** Inhibiting the apoptosis pathway upstream or downstream of MOMP in Jurkat cells by generating Caspase-9 knockout and BAX/BAK double knockout cell lines does not protect from Raptinal-induced cell death at longer time points. **B** In addition to its primarily caspase-dependent mechanism of cell death, Raptinal can induce caspase-independent cell death from mitochondrial damage. Panel **A** was reproduced from Heimer et al. [35] copyright 2019 CC BY 4.0. Panel **B** was recreated from Heimer et al. [35] copyright 2019 CC BY 4.0 and created with BioRender.com.

The initial studies of Raptinal’s MOA [34, 35] and subsequent experiments utilizing Raptinal establish apoptosis as the primary mode of cell death induced by Raptinal. However, it was also shown that Raptinal’s disruption of general mitochondrial function due to MOMP causes cell death in a caspase-independent manner [35]. Caspase-9 knockout protects from cell death at short timepoints, but at longer timepoints it is unable to protect from cell death (Fig. 8A) [35]. As expected, BAX/BAK DKO had no effect on Raptinal cytotoxicity in both the short and long incubations. These experiments suggest that general disruption of mitochondrial activity by Raptinal can be enough to induce cell death at longer time points through a caspase-independent mechanism (Fig. 8B) [35].

### Pyroptosis induced by Raptinal

**Fig. 9:**
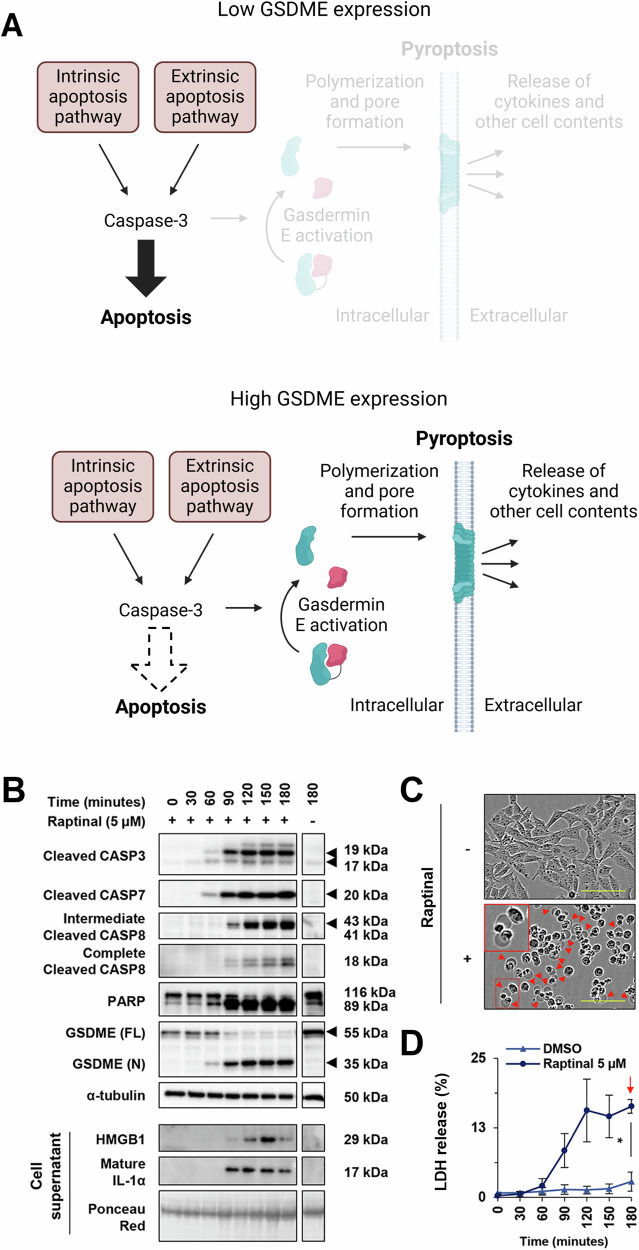
Raptinal can induce caspase-3 dependent pyroptosis. **A** Caspase-3 can cleave gasdermin E and promote pyroptotic cell death when cell express high levels of GSDME. **B** Raptinal (5 μM) activated caspase-3 can cleave GSDME in A375 cells. Raptinal (5 μM) can induce GSDME-dependent pyroptosis in A375 cells as seen by **C** cell death morphology (scale bar 100 μm) and **D** LDH release. Panel **A** was created with BioRender.com. Panels **B**–**D** were reproduced from Vernon et al. [36] Reprinted with permission from American Association for Cancer Research, copyright 2022.

There are specific conditions where Raptinal-induced cell death remains caspase dependent yet deviates from apoptosis. Pyroptosis is a lytic form of cell death that typically involves inflammasome activation and release of inflammatory cytokines through pores formed by gasdermin proteins [9, 129]. Raptinal was instrumental in demonstrating that expression levels of gasdermin-E (GSDME) determine if activation of caspase-3 leads to apoptosis or pyroptosis [36]. Cleavage of full-length GSDME allows the N-terminal portion to polymerize to form a pore in the membrane, leading to the immunogenic cell death termed pyroptosis (Fig. 9A) [36]. It was known that granzyme B could cleave GSDME to lead to pyroptosis [46], but this new study revealed that caspase-3 could also cleave GSDME (Fig. 9B) [36]. Cells expressing GSDME and treated with Raptinal also show morphological features of pyroptosis and release LDH (Fig. 9C, D). Thus, in cells expressing high levels of GSDME, Raptinal (and other pro-apoptotic compounds) may also induce death by pyroptosis through the activation of caspase-3 and subsequent cleavage of GSDME.

## Raptinal as a tool compound in the study of pyroptosis

The ability of Raptinal to induce pyroptotic cell death in GSDME expressing cells has allowed Raptinal to be used as a tool to further understand pyroptosis. As previously mentioned, the pyroptosis-inducing feature of Raptinal was instrumental in discovering the link between caspase-3 activated GSDME and pyroptosis [36]. Further evaluation of the role of GSDME in pyroptosis used Raptinal in a CRISPR screen for proteins important in Raptinal-induced pyroptosis in HeLa cells, and these experiments identified USP48 as a critical protein to pyroptotic cell death, and its expression correlates with effectiveness of PD-1 therapy, in a manner also dependent on GSDME expression [130].

**Fig. 10:**
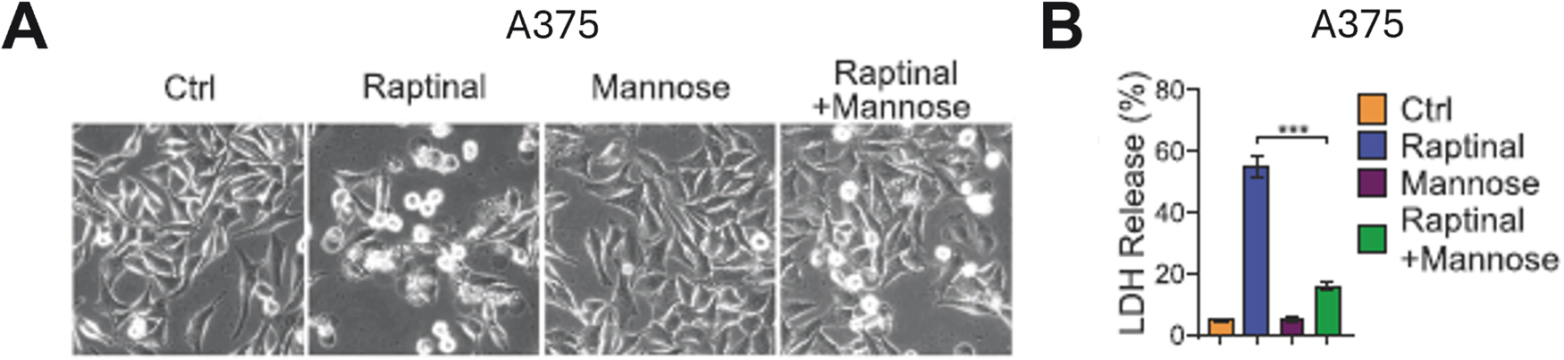
Raptinal as a tool compound to explore pyroptosis. **A** Mannose (20 mM, 2 h pretreatment) protects from Raptinal-induced pyroptosis in A375 cells with 24 h treatment with 1 μM Raptinal. **B** LDH release from Raptinal-treated A375 cells from **A** is inhibited by cotreatment with mannose. Panels **A** and **B** were reproduced from Ai et al. [131] copyright 2023 CC BY 4.0.

While there may be immunological benefits of inducing pyroptosis in tumors, GSDME is normally expressed at higher levels in healthy tissue compared to cancer cells, and chemotherapies that kill cancer by apoptosis often have off-target toxicities because activated caspase-3 can cleave GSDME, leading to pyroptosis in healthy cells [131]. Mannose has been shown to reduce off-target toxicities of chemotherapy treatment, demonstrated by its ability to protect A375 cells from Raptinal-induced pyroptosis (Fig. 10A, B) [131]. Raptinal was used to elucidate the mechanism of this protective effect of mannose: activating the AMPK and hexosamine biosynthetic pathway and ultimately leading to higher phosphorylation of GSDME, resulting in the inability of caspase-3 to cleave GSDME and induce pyroptosis [131]. Thus, if a tumor were to be treated by Raptinal or other apoptosis inducer, mannose could be a potential cotreatment to protect healthy cells from undesired pyroptosis while still inducing apoptosis in the tumor.

Recent reports of the more established GSDMD-mediated pyroptosis routes have also benefitted from using Raptinal as an orthogonal inducer of pyroptosis. In innate immune cells, pathogen-associated molecular pattern molecules (PAMPs) or other signals activate the inflammasome, which activates caspase-1 and, consequently, GSDMD and cytokines like IL-1β and IL-18; and GSDMD forms the membrane pores responsible for pyroptosis. Irregularities in this pathway can have many implications in immunoinflammatory diseases. In caspase-1 deficient conditions, cell death and release of cytokines is delayed but still observed [123]. While this cell death does involve caspases, it does not elicit morphologically apoptotic cell death phenotypes in comparison to Raptinal and doxorubicin [123]. Thus, in the absence of caspase-1 or GSDMD, there can still be an alternative lytic form of cell death, and any attempts to prevent cell death should aim to prevent inflammasome formation instead of the subsequent diverging pathways [123]. Similarly, it has been shown that when the NLRP3 inflammasome is constitutively active, there is a means of GSDMD independent pyroptosis that (similar to Raptinal-treated cells) instead relies on GSDME-mediated pyroptosis [132]. Since Raptinal can also induce GSDME-mediated pyroptosis, it was a helpful control to study inhibition of pyroptosis in these various contexts.

In addition to its roles in studying apoptosis and pyroptosis, Raptinal has been useful as a control in probing cell death that does not fit neatly into either mode of cell death. It was found that activators of the NLRP3 inflammasome can cause lytic cell death that was not strictly pyroptotic or apoptotic [133]. Elsewhere, while some compounds were found to kill by apoptosis in normoxia but by necrosis in hypoxia, Raptinal served as a control that was apoptotic in both normoxic and hypoxic conditions, though faster in hypoxia [49].

## Raptinal’s translational potential

**Fig. 11:**
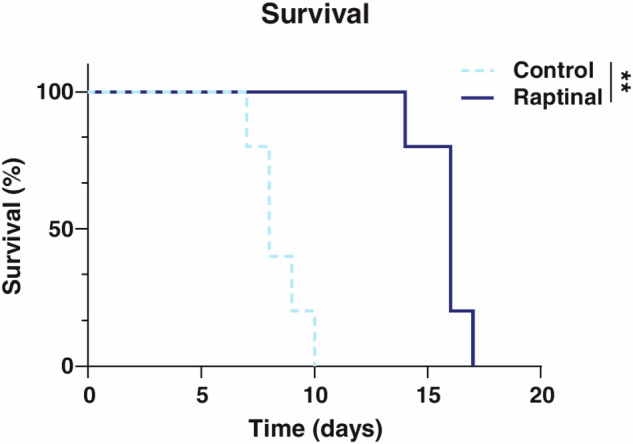
Extended survival was observed in C57BL/6J mice with subcutaneous YUMM1.7 tumors that were treated by intraperitoneal injection with vehicle or 20 mg/kg Raptinal once-per-day for four days. Reproduced from Vernon et al. [36] Reprinted with permission from American Association for Cancer Research, copyright 2022.

While Raptinal’s translational potential is currently underexplored, this compound may offer some advantage over certain chemotherapies. For instance, if a tumor develops resistance due to some alteration early in the apoptosis pathway, Raptinal may be an option to trigger cell death downstream of these apoptotic defects. Some proapoptotic drugs have limitations in dosing, such as doxorubicin’s cardiotoxicity that limits its cumulative lifetime dose, and developing alternative treatments is essential. Thus far, Raptinal has been shown to have some efficacy in vivo in both a murine melanoma and breast cancer tumor model when dosed 20 mg/kg once daily for 3 or 4 consecutive days [34]. Raptinal was also shown to delay tumor growth and lengthen overall survival in C57BL/6J mice with YUMM1.7 tumors treated with Raptinal at 20 mg/kg (Fig. 11) [36]. There were signs of toxicity as seen by initial weight loss, but this weight was regained after treatment [36]. It should be noted that it is currently unclear if Raptinal can selectively target cancer cells over normal cells; any specificity could become more apparent once the exact molecular target of Raptinal is elucidated.

Raptinal has been radiolabeled by ^99m^Tc so its biodistribution could be tracked in rats and its efficacy observed in an HCC model [126]. Nonspecific protein binding led to high retention in the blood in both healthy rats and those bearing HCC tumors; but there was higher retention in the liver of those bearing an HCC tumor than those without, suggesting a small preference for tumoral tissue [126]. However, labeling Raptinal may disrupt its biodistribution; as such, label-free methods of monitoring the distribution of Raptinal would be ideal. In attempts to gain better selectivity and efficacy, Raptinal silver nanoparticles have also been generated [134]. Treatment shows cell death in HCC tumors and improved liver enzyme levels; however, they do not present data on overall tumor reduction over time [134]. This early work in testing Raptinal in vivo suggests it may find translation applications, but minimizing toxicity will be critical.

Recent advances in targeted drug delivery may prove applicable to Raptinal. Passive targeting using carriers such as nanoparticles or liposomes could be leveraged for their ability to accumulate in tumors over healthy tissue. More selective targeting could employ antibody-drug conjugates to bind specific cells or a prodrug approach where an inactivating group would be cleaved within the cancer cell or tumor microenvironment. Exploring these methods using Raptinal may prove useful in minimizing toxicity.

Evasion of apoptosis is observed in certain autoimmune diseases and viral infections [135, 136]. Accordingly, chemotherapies have been investigated for treating a wide range of autoimmune diseases [137], and Raptinal could be explored as another option. In some cases, viruses may inhibit apoptosis in infected cells, allowing more opportunity for viral replication. For instance, adenovirus infection can involve the expression of the E1A gene to promote DNA replication and the E1B 19K protein to inhibit apoptosis by inhibiting BAX [138]. Thus, if Raptinal could be directed to infected cells, it could bypass the E1B 19K inhibited BAX and be utilized to effectively kill these cells and diminish viral replication. While not for therapeutic benefit, Raptinal was also used in a study exploring contributing factors to postoperative cognitive dysfunction [139]. In this study, it was found that anesthesia and laparotomy in mice activated the cGAS/STING pathway and subsequent caspase-3/GSDME pyroptosis within the hippocampus, leading to poorer performance in cognitive tests. Treatment with a cGAS inhibitor before surgery could reverse these effects, but Raptinal (20 mg/kg, intraperitoneal injection, 30 min before surgery), which can cause GSDME pyroptosis independent of cGAS, reversed the protective effects of the cGAS inhibitor [139]. While treatment with Raptinal was only shown to reduce the protective effects of cGAS inhibitor and implications of single Raptinal treatment are not shown, these results present more off target effects to consider when exploring Raptinal’s translational potential and should be investigated further.

## Conclusion and future directions

The ability of Raptinal to rapidly induce MOMP and release of cytochrome c downstream of BAX/BAK activation has made this compound useful in 1) studying specific apoptosis events and, 2) providing a convenient and reliable apoptotic/cell death reference compound. Additionally, the discovery of the ability of caspase-3 to activate GSDME and induce pyroptosis was not only a significant addition to Raptinal’s MOA, but it was also important for fundamental understanding of pyroptosis.

The exact molecular target of Raptinal remains unknown; given its unique rapid-acting phenotype, the target of Raptinal might be expected to be different from other pro-apoptotic compounds. Identification of this target may reveal yet another layer of Raptinal’s MOA and further elucidate mechanistic details of apoptosis. Chemical proteomic techniques could be leveraged to explore Raptinal’s interactions with proteins within the cell. If tolerated sites for modification can be established, probes could be generated for various binding, pulldown, or colocalization assays. To explore the structure-activity relationship (SAR) of Raptinal, some structural modifications have previously been made, but these modifications all led to reduced activity [34]. While modifications of Raptinal’s dialdehyde moiety led to complete loss of activity, minor modifications of the aryl rings were tolerated but did result in compounds with reduced potency. Raptinal’s SAR could also help elucidate mechanisms of inhibition of PANX1 activity, and structural modifications could be assessed based on their potency and selectivity toward inducing apoptosis vs inhibiting PANX1 activity, further enlightening our understanding of Raptinal’s MOA.

The few in vivo studies with Raptinal show that there is promise for leveraging this compound in treating tumors, but further studies on its efficacy and toxicity are needed. The traits that make Raptinal an outstanding tool compound (rapid and quantitative induction of cell death in virtually any cell line) may not bode well for its translational advancement. In this context, methods of targeted drug delivery, such as antibody drug conjugates or direct intratumoral injections, may enable the potency of Raptinal to be harnessed in a more selective manner. Alternatively, further developing mannose (or other compounds) as a protective cotreatment with Raptinal may improve the observed toxicity effects. While the promising results of Raptinal warrant further investigation of its translational potential, there is little question about its importance as a tool in cell death research.
